# Perceived Workplace Stress Is Associated with an Increased Risk of Prostate Cancer before Age 65

**DOI:** 10.3389/fonc.2017.00269

**Published:** 2017-11-13

**Authors:** Audrey Blanc-Lapierre, Marie-Claude Rousseau, Marie-Elise Parent

**Affiliations:** ^1^Epidemiology and Biostatistics Unit, INRS-Institut Armand-Frappier, Institut National de la Recherche Scientifique (INRS), University of Quebec, Laval, QC, Canada; ^2^School of Public Health, Department of Social and Preventive Medicine, University of Montreal, Montreal, QC, Canada; ^3^University of Montreal Hospital Research Center (CRCHUM), Montreal, QC, Canada

**Keywords:** psychological stress, workplace, prostate cancer, case–control study, occupational health

## Abstract

**Background:**

Evidence is lacking regarding the potential role of chronic psychological stress on cancer incidence. The workplace is reported to be the main source of stress among Canadian men. We examined the association between perceived lifetime workplace stress and prostate cancer (PCa) risk in a large case–control study.

**Methods:**

Cases were 1,933 men, aged ≤ 75 years, newly diagnosed with PCa in 2005–2009 across hospitals in Montreal, Canada. Concurrently, 1994 population controls frequency-matched on age were randomly selected from the electoral list based on cases’ residential districts. Detailed lifestyle and work histories (including perceived stress, from any type of work stressor, for each job held) were collected during in-person interviews. Logistic regression was used to estimate odds ratios (OR) and 95% confidence intervals (CI) for the association between work-related stress and PCa risk in multivariate analyses.

**Results:**

Over the lifetime, 58% of subjects reported at least one job as stressful. Occupations described as stressful were most often among white-collar workers. Perceived workplace stress duration was associated with a higher risk of PCa (OR = 1.12, 95% CI:1.04–1.20 per 10-year increase) among men younger than 65 years, but not among older men. Associations were similar irrespective of PCa aggressiveness. Frequent or recent screening for PCa, age at first exposure and time since exposure to work-related stress, and socioeconomic and lifestyle factors, had little influence on risk estimates.

**Conclusion:**

Findings are in line with an association between reporting prolonged workplace stress and an increase in risk of PCa before age 65.

## Introduction

Chronic psychological stress has been found to have a deleterious impact on health, increasing the risk of cardiovascular disease (1), hypertension (2), mental illness (3), and diabetes (4). Its role in cancer etiology is of growing interest and was earmarked as a high-priority exposure for review by the International Agency for Research on Cancer (5). Chronic stress may have an impact on cancer development *via* the activation of the sympathetic nervous system and the hypothalamic–pituitary–adrenal axis (6). Most studies to date have been conducted on breast cancer risk (7–9); only four focused on prostate cancer (PCa) risk (10–13). Few of them assessed chronic stress at work. The workplace is the main source of stress among Canadian workers, particularly in men (14). Previous studies have most often assessed work-related stress using the Karasek model focusing on job strain, defined as an imbalance between high demand and low control (15). However, this objective measure may not correspond to the perception of stress by the subject and does not account for sources of stress other than job strain in the workplace. It has been advanced that the perception of threat from stressors may in fact be key in determining the behavioral and physiologic responses to stress (6, 16, 17).

A major issue to previous studies is that their assessment of stress was most often limited to one (usually at study baseline) or two time point(s), thereby rarely capturing changes in stress level over the entire career. We present here findings on the association between perceived stress, from any type of job stressor and over the entire work history, and PCa risk.

## Materials and Methods

### Study Population

The Prostate Cancer & Environment Study (PROtEuS) is a population-based case–control study conducted in Montreal, Canada, to assess the role of environmental factors in the development of PCa. In brief, eligible subjects were men, younger than 76 years of age at the time of diagnosis or selection, residents of the greater Montreal area, registered on Quebec’s permanent electoral list. In order to comply with institutional regulations and insure comprehensive population coverage at recruitment, the study base was restricted to men who referred or would be expected to refer to a French hospital for a PCa diagnosis. This represents the vast majority of Montreal residents as according to census data, French was the language used at home by 76% of Montrealers in 2006.

Cases were all patients newly diagnosed with primary histologically confirmed PCa, actively ascertained through pathology departments across French hospitals which diagnose and treat PCa in the Montreal area between 2005 and 2009. This covered over 80% of all PCa cases diagnosed in the region of Montreal during the study period according to registry information. There are no specific reference hospitals for PCa in Montreal. Concurrently to case recruitment, controls were randomly selected from the electoral list of French-speaking men residing in the same districts as cases and frequency matched to cases by 5-year age groups. For 3% of cases and 4% of controls, interviews were conducted with a proxy, usually the spouse.

Study participants (1,933 cases and 1,994 controls) represented 79% of eligible cases and 56% of eligible controls.

This study was approved by the Ethics Committees of the following institutions: Institut national de la recherche scientifique, Centre de Recherche du Centre Hospitalier de l’Université de Montréal, Hôpital Maisonneuve-Rosemont, Hôpital Jean-Talon, Hôpital Fleury, and Hôpital Charles-LeMoyne. The study was carried out in compliance with their recommendations. All subjects provided written informed consent in accordance with the Declaration of Helsinki.

### Data Collection

During face-to-face interviews, subjects provided information on socio-demographic characteristics, lifestyle (including smoking habits, alcohol consumption, and dietary habits) and medical history (including depression and treatment with medication, family history of cancer, and PCa screening). A detailed occupational history, including workplace physical activity and perceived stress at work, was collected.

Job categories were defined according to the Canadian Classification and Dictionary of Occupations. We used socioeconomic classifications of occupations from the Euro ESeC matrix (employment relation: employee/self-employed/employer; socioeconomic position: salariat/intermediate/working class) (18) and from Pineo et al. (“white-collar” jobs: professional and high-level managerial/semi-professional, technical and middle managerial/supervisors and foremen; “blue-collar” jobs: skilled workers and employees/semi-skilled workers and employees/unskilled workers and employees) (19). The degree of aggressiveness of PCa, defined by the Gleason score, was extracted from pathology reports.

### Workplace Stress Assessment

A primary focus of the study was the assessment of occupational chemical exposures. Toward this, trained interviewers elicited detailed descriptions of each job held for at least 2 years over the lifetime, including tasks, equipment, and products used, work environment and conditions. This often required 1–2 h. Toward the end of questioning about each job, participants were asked “Did this job make you feel tense, anxious, or stressed out most of the time?” This question was used to define perceived workplace stress for a given job, based on a dichotomous response (yes/no).

### Statistical Analyses

Analyses were restricted to participants who had completed the occupational questionnaire for at least one job. The distribution of stressful jobs was described according to job characteristics and occupational categories.

Unconditional logistic regression was used to assess the risk of PCa associated with perceived workplace stress, using men who had never experienced job stress as the reference. Odds ratios (ORs) for low-grade (Gleason scores <7 or [3 + 4]) and high-grade (Gleason scores > 7 or [4 + 3]) PCa (20) were estimated in polynomial logistic models, and their respective regression coefficients were compared using a Wald test.

Workplace stress was assessed based on the cumulative number of years in jobs reported as stressful over the lifetime. Work stress duration was entered in the model as a quantitative variable (after confirming linearity of the logit of PCa risk) or categorized as follows: 0, ]0–15], ]15–30], and >30 years, whereas exposed categories were approximate tertiles of the distribution among controls in the main analyses.

Two regression models were built, the first adjusting for age only (in years, continuous), the second also including other factors known or suspected to be associated with PCa diagnosis: ancestry (European/Sub-Saharan/Asian/Greater Middle East/Other/Do not know), first-degree family history of PCa (Yes/No/Do not know), family income (<$C30,000/$C30,000–79,999/$C80,000 and more/Preferred not to respond/Do not know), education (Primary or secondary/College or university), marital status (Married or common law/Separated or divorced/Single/Widower), body mass index (kg/m^2^, continuous), type 2 diabetes (Yes/No), depression treated with medication (Yes/No), alcohol consumption (drink-years), smoking (pack-years), physical activity at work (in Metabolic equivalents—hours/day), and dietary habits (frequency of fruit and vegetable intake). There were no missing data for age, family history of PCa, type 2 diabetes, and depression treated with medication. For other variables, the numbers of missing data were as follows (numbers in parentheses): family income (2), education (7), marital status (1), physical activity at work (3), BMI (23), alcohol consumption (118), smoking (30), and frequency of fruit and vegetable intake (46). Terms indicating missing data for each covariate were entered in the models. Effect of additional adjustment for PCa screening frequency [≥ 5 prostatic specific antigen (PSA) tests and/or digital rectal exam (DRE) tests during the previous 5 years: Yes/No] was examined.

Different analyses were carried out to evaluate whether the timing of exposure to stress had an influence on PCa risk. We investigated if the association between work-related stress duration and PCa risk differed according to age at first stress experience. We also examined if this association was impacted by the time elapsed (</≥5 years) between the last stress exposure and the index (diagnosis/interview) date. In addition, we applied a lag time of 5 years preceding the index date to take into consideration a possible latency of effect. We also tested whether the occupational socioeconomic class (White-collar/Blue-collar workers), age (</≥ age 65) or retirement at index date modified the association between workplace stress and PCa. Indeed, age and occupational classes have been suggested to modulate the relationship between job stress and well-being (21) or cardiovascular diseases (22, 23). Effect modification was examined considering either the *P*-value of the likelihood ratio test comparing models before and after inclusion of product interaction terms, or the *P*-value for any of the individual product terms.

Sensitivity analyses were performed restricting to subjects with available information on workplace stress for each job or to subjects screened for PCa (PSA or DRE) within 2 years of the index date. We also performed analyses stratified according to any experience of night work, defined as at least one job including three working hours between midnight and 5 a.m. Finally, we examined if restricting analyses to self-respondents or to subjects without treatment for depression had an impact on the results.

All analyses were performed using SAS software (9.3; SAS Institute Inc., Cary, NC, USA).

## Results

The study population for analyses comprised 1,931 cases (including 538 high-grade PCa) and 1,991 controls. Compared to subjects included in the analyses, the 202 subjects who were excluded because of incomplete occupational questionnaires were older (66.0 vs 64.1 years), more often proxy respondents (9.4 vs 3.1%), less educated (37.2 vs 48.8% with a post-secondary level), had a lower family income (33.0 vs 24.6% with an income <$C30 000) and reported higher levels of daily physical activity at work (2.7 vs 2.5 METS-hours). They were comparable according to other characteristics. Cases were slightly younger (64 vs 65 years, on average) and less educated than controls (Table 1). As expected, cases were more likely than controls to have a family history of PCa, to be of Sub-Saharan ancestry and to have been screened for PCa in the last 2 years. They were less likely to be of Greater Middle East or Asian ancestry. Annual family income, marital status, and socioeconomic class of occupations (Professional/Non-professional) did not differ according to case–control status. Cases and controls were similar in terms of workplace physical activity, fruit and vegetable consumption, smoking habits, and alcohol consumption. Average BMI was slightly lower and type 2 diabetes less frequent among cases.

**Table 1:** Selected characteristics of participants.

	Controls (*n* = 1,991)		Cases (*n* = 1,931)		
Characteristics	*n*	%	*n*	%	*P*-value
**Proxy respondent**	76	3.8	58	3.0	0.16
**Ancestry**					
European	1,683	84.5	1,691	87.6	<0.01
Sub-Saharan	90	4.5	129	6.7	
Asian	72	3.6	24	1.2	
Greater Middle East	99	5.0	45	2.3	
Other (Hispanics, Aboriginals)	33	1.7	30	1.6	
Do not know	14	0.7	12	0.6	
**Annual family income**					0.13
<$C10 000–$C29 999	495	24.9	488	25.3	
$C30 000–$C79 999	872	43.8	871	45.1	
$C80 000–>$C100 000	428	21.5	425	22.0	
Preferred not to respond/do not know	194	9.8	147	7.6	
**Marital status**					0.47
Married or common law	1,501	75.4	1,428	74.0	
Separated or divorced	249	12.5	256	13.2	
Single	157	7.9	174	9.0	
Widower	83	4.2	73	3.8	
**Education**					0.23
Primary	427	21.5	447	23.2	
Secondary	578	29.0	576	29.9	
College/University	984	49.5	903	46.9	
**Socioeconomic position^a^**					0.28
Blue collar^b^	1,066	53.6	1,067	55.3	
White collar	923	46.4	862	44.7	
**Last PCa screening (PSA and/or DRE)**					<0.01
≤2 years	1,509	75.8	1,911	99.0	
>2 years	234	11.7	1	0.0	
No	191	9.6	3	0.2	
Do not know	57	2.9	16	0.8	
**First-degree family history of PCa**					<0.01
No	1,737	87.3	1,415	73.3	
Yes	198	9.9	450	23.3	
Do not know	56	2.8	66	3.4	
**Type 2 diabetes**	347	17.4	205	10.6	<0.01
**Treated depression**	181	9.1	221	11.5	<0.01
**Work-related stress (years)**					<0.01
No	879	44.1	785	40.6	
]0–15]	320	16.1	289	15.0	
]15–30]	356	17.9	435	22.5	
>30	436	21.9	422	21.9	

	**Mean**	**SD**	**Mean**	**SD**	

Age at index date (years)	64.8	6.9	63.6	6.8	
Daily physical activity at work (METs—hours)^c^	2.5	0.9	2.5	1.0	0.55
Daily frequency of fruits and vegetables consumption	4.5	2.1	4.4	2.1	0.70
Smoking (pack-years)	24.2	27.9	22.9	27.6	0.15
Alcohol intake (drink-years)	73.9	136.6	75.2	121.5	0.75
Body mass index (kg/m^2^)	27.2	4.4	26.7	4.0	<0.01

*PCa, prostate cancer; PSA, prostate-specific antigen; DRE, digital rectal examination; METs, metabolic equivalents*.

*^a^According to Pineo et al. (19)*.

*^b^Held blue-collar jobs during most of their occupational life*.

*^c^Mean of metabolic equivalents assessed for each task during occupational life, weighted by duration of tasks and jobs*.

On average, cases and controls had held 4.9 (SD: 2.6, range: 1–20) and 5.2 (SD: 2.7, range: 1–24) jobs over their lifetime, respectively. Subjects reporting at least one job as stressful represented 59.4% of cases (*N* = 1,146) and 55.8% of controls (*N* = 1,112). Perceived stress was less frequently reported by proxy respondents (50.0%). Overall, the mean cumulative duration of stressful jobs was of 24.8 (SD: 12.4) years. Subjects ever stressed by their job tended to be younger and more frequently treated for depression (data not shown). They were also more likely to be employed in white-collar occupations (52.0 vs 36.9% among never exposed), to have a higher annual family income and education, and lower levels of physical activity at work, and of tobacco and alcohol consumption (data not shown).

When comparing non-stressful jobs to stressful ones (Table 2), the latter were characterized by less exposure to hazards or noise/vibration, less physical or manual activities, increased socioeconomic position, and increased number of hours worked per week. Stressful jobs occurred more recently (≥1980), and more often in the middle of the career (at ages 30–50 years).

**Table 2:** Characteristics of stressful and non-stressful jobs.

	Non-stressful jobs (*N* = 9,118)		Stressful jobs (*N* = 5,250)		
	*N*	Percent	*N*	Percent	*P*-value
Hazards^a^	2,269	24.9	972	18.5	<0.01
Noise and/or vibration^b^	1,733	19.0	767	14.6	<0.01
Frequent lifting and/or carrying of heavy objects (>10 lbs)	3,515	43.2	1,291	28.9	<0.01
**Physical activity level (METs/day)**					
<1.5	419	4.6	242	4.6	
1.5–3	5,691	62.3	3,881	73.8	
3–6	2,716	29.7	1,072	20.4	
>6	309	3.4	62	1.2	
**Worker functions-functional relationship to:**					
**a.Data**					<0.01
Data compiling, computing, copying, or comparing	4,520	53.0	1,799	36.8	
Data synthesizing, coordinating, or analyzing	3,929	46.1	3,068	62.7	
No relationship	81	0.9	25	0.5	
**b.People**					<0.01
Mentoring, negotiating, instructing, supervising, diverting, persuading, speaking-signaling, or serving (relationship to people)	6,047	70.9	3,911	80.0	
No relationship	2,483	29.1	981	20.0	
**c.Things**					<0.01
Setting-up, precision working, operating-controlling, driving operating, manipulating-operating, tending, feeding-offbearing or handling (relationship to things)	5,925	69.5	2,640	54.0	
No relationship	2,605	30.5	2,252	46.0	
**Employment relation (Euro ESeC^c^)**					<0.01
Employee	7,211	79.7	3,593	69.2	
Self-employed	375	4.2	284	5.5	
Employer	3	16.1	1,317	25.3	
**Socioeconomic position (Euro ESeC^c^)**					<0.01
Salariat	2,587	28.6	2,073	39.9	
Intermediate	1,519	16.8	940	18.1	
Working class	4,941	54.6	2,181	42.0	
**Socioeconomic position [Pineo (19)^d^]**					<0.01
Professional or “white-collar” jobs	5,711	62.5	2,394	45.5	
Non-professional or “blue-collar” jobs	3,424	37.5	2,863	54.5	
**Number of hours worked per week**					<0.01
< 41	6,227	68.3	2,947	56.2	
≥ 41	2,892	31.7	2,295	43.8	
**Number of weeks worker per year**					0.31
<48	1,800	19.7	998	19.0	
≥ 48	7,330	80.3	4,250	81.0	
**Work era**					<0.01
<1980	4,903	53.7	2,021	38.4	
≥1980	4,232	46.3	3,236	61.6	
**Timing of job during the occupational life**					<0.01
Early during career (age<30)	3,576	40.0	1,230	24.0	
Middle of career (age 30–50)	3,701	41.4	2,812	54.7	
End of career (age≥50)	1,667	18.6	1,094	21.3	

*METs, metabolic equivalents*.

*^a^Situations in which the individual was exposed to the definite risk of bodily injury*.

*^b^Sufficient noise, either constant or intermittent, to cause marked distraction or possible injury to the sense of hearing*.

*^c^According to the European socio-economic classification (Euro ESeC matrix)*.

*^d^According to Pineo et al. (19): “white-collar” jobs: professional and high-level managerial/semi-professional, technical and middle managerial/supervisors and foremen; “blue-collar” jobs: skilled workers and employees/semi-skilled workers and employees/unskilled workers and employees*.

The job categories with the greater proportions of stressful jobs (55% of all jobs within a category) were Management occupations, transport and communications operations; Supervisors, food and beverage preparation and related service occupations, and; Lawyers and notaries (Table 3).

**Table 3:** Job title categories with proportions of stressful jobs >40%.

CCDO code^a^	Job title category	*n* total^b^	% of stressful jobs
1147	Management occupations, transport, and communications operations	56	55
6120	Supervisors, food and beverage preparation, and related service occupations	107	55
2343	Lawyers and notaries	91	55
1149	Other managers and administrators	243	54
1135	Financial management occupations	205	54
8580	Foremen, mechanics, and repairers, except electrical	76	53
1137	Sales and advertising management occupations	188	52
1113	Government administrators	65	51
1130	General managers and other senior officials	203	51
3330	Producers and directors, performing and audiovisual arts	56	50
1145	Management occupations, construction operations	60	48
5171	Insurance sales occupations and agents	85	48
3351	Writers and editors, publication	52	48
2143	Civil engineers	129	48
9173	Taxi drivers and chauffeurs	136	46
2731	Elementary and kindergarten teachers	103	45
1143	Production management occupations	108	44
6165	Pressing occupations	55	44
5131	Technical sales occupations and related advisers	113	43
5130	Supervisors: sales, occupations, commodities	397	43
9512	Printing press occupations	63	43
2183	Systems analysts, computer programmers, and related occupations	164	43
1131	Management occupations: natural sciences, engineering, and mathematics	113	42
2711	University teachers	198	42
1179	Occupations related to management and administration	189	42
5172	Real estate sales occupations	86	42
414	Office machine and electronic data-processing equipment operators	54	41
4130	Supervisors, bookkeeping, account-recording and related occupations	52	40
2144	Electrical engineers	72	40

*^a^CCDO, Canadian Classification and Dictionary of Occupations*.

*^b^For jobs with n > 50 in our study population*.

As there appeared to be some interaction between workplace stress duration (categorical variable) and age at index date (</≥ age 65, *p* = 0.06), the remaining sets of results were stratified by age.

Among subjects aged 65 or more, PCa risk was not associated with perceived workplace stress duration (Table 4). By contrast, among subjects younger than age 65, a dose–response trend was observed between PCa risk and stress duration categories (*P* trend <0.01, Table 5). PCa risk was elevated among younger men reporting more than 30 years of workplace stress; risk increased linearly with workplace stress duration (OR = 1.12, 95% CI: 1.04–1.20 per 10-year increment). Low-grade and high-grade PCa followed generally similar patterns of risk. Models adjusting for age only or for several potential socio-demographic and lifestyle confounders yielded similar risk estimates.

**Table 4:** OR for the risk of PCa associated with the duration of perceived workplace stress among subjects aged 65 or more at diagnosis or interview.

	PCa risk						
		Age-adjusted model			Multivariate model^a^		
Exposure to workplace stress	*n* cases	OR	95% CI	*P* for trend	OR	95% CI	*P* for trend
**All subjects**							
Any exposure	475	0.97	0.81–1.15		0.99	0.82–1.20	
Cumulative duration (years)				0.70			0.96
]0–15]	107	0.82	0.62–1.09		0.85	0.63–1.14	
]15–30]	165	1.28	0.99–1.65		1.28	0.98–1.68	
>30	203	0.87	0.70–1.09		0.91	0.72–1.15	
**Low-grade PCa cases^b^**							
Any exposure	325	0.97	0.80–1.18		0.98	0.80–1.21	
Cumulative duration (years)				0.87			0.98
]0–15]	69	0.78	0.56–1.07		0.79	0.57–1.12	
]15–30]	115	1.31	0.98–1.73		1.30	0.96–1.75	
>30	141	0.89	0.69–1.14		0.91	0.70–1.18	
**High-grade PCa cases^b^**							
Any exposure	150	0.96	0.74–1.25		1.02	0.78–1.33	
Cumulative duration (years)				0.57			0.92
]0–15]	38	0.92	0.62–1.38		0.97	0.64–1.47	
]15–30]	50	1.22	0.84–1.76		1.25	0.85–1.84	
>30	62	0.84	0.60–1.17		0.92	0.65–1.29	
**Subject screened for PCa in the last 2 years**							
Any exposure	474	0.94	0.78–1.13		0.99	0.81–1.20	
Cumulative duration (years)				0.55			0.99
]0–15]	107	0.79	0.59–1.05		0.82	0.60–1.11	
]15–30]	164	1.27	0.97–1.67		1.31	0.98–1.74	
>30	203	0.85	0.67–1.07		0.91	0.71–1.17	
**5-year lag time**							
Any exposure	475	0.97	0.81–1.16		1.00	0.83–1.20	
Cumulative duration (years)				0.55			0.78
]0–15]	112	0.85	0.64–1.12		0.88	0.66–1.18	
]15–30]	179	1.29	1.01–1.66		1.31	1.01–1.70	
>30	184	0.83	0.66–1.05		0.87	0.68–1.11	
**Retired subjects**							
Any exposure	357	0.91	0.74–1.12		0.92	0.74–1.15	
Cumulative duration (years)				0.56			0.68
]0–15]	82	0.80	0.58–1.11		0.81	0.58–1.14	
]15–30]	119	1.08	0.80–1.45		1.09	0.80–1.50	
>30	156	0.88	0.68–1.15		0.90	0.69–1.19	
**Self-respondents**							
Any exposure	459	0.97	0.81–1.17		1.00	0.83–1.21	
Cumulative duration (years)				0.78			0.98
]0–15]	104	0.82	0.62–1.09		0.85	0.63–1.14	
]15–30]	161	1.29	0.99–1.67		1.29	0.98–1.70	
>30	194	0.88	0.70–1.11		0.91	0.72–1.16	

*OR, odds ratio; CI, confidence interval; PCa, prostate cancer*.

*^a^Adjusted for age, ancestry, first-degree family history of PCa, family income, education, marital status, body mass index, type 2 diabetes, depression treated with medication, alcohol consumption, smoking, physical activity at work, and frequency of fruit and vegetable intake*.

*^b^Polynomial logistic regression model. Reference category: subjects reporting no perceived work-related stress*.

**Table 5:** Odds ratios (OR) for the risk of PCa associated with the duration of perceived workplace stress among subjects younger than age 65 at diagnosis or interview.

	PCa risk						
		Age-adjusted model			Multivariate model^a^		
Exposure to workplace stress	*n* cases	OR	95% CI	*P* for trend	OR	95% CI	*P* for trend
**All subjects**							
Any exposure	671	1.24	1.02–1.49		1.25	1.02–1.52	
Cumulative duration (years)				0.01			0.01
]0–15]	182	1.05	0.81–1.37		1.06	0.81–1.40	
]15–30]	270	1.31	1.03–1.66		1.28	0.99–1.64	
>30	219	1.33	1.03–1.71		1.40	1.07–1.82	
**Low-grade PCa cases^b^**							
Any exposure	507	1.22	1.00–1.49		1.23	0.99–1.52	
Cumulative duration (years)				0.01			0.01
]0–15]	134	1.01	0.76–1.34		1.01	0.75–1.36	
]15–30]	212	1.34	1.04–1.72		1.31	1.00–1.71	
>30	161	1.29	0.99–1.69		1.36	1.02–1.80	
**High-grade PCa cases^b^**							
Any exposure	164	1.28	0.95–1.73		1.31	0.96–1.78	
Cumulative duration (years)				0.07			0.05
]0–15]	48	1.20	0.80–1.80		1.24	0.82–1.88	
]15–30]	58	1.20	0.82–1.76		1.18	0.79–1.75	
>30	58	1.45	0.99–2.14		1.53	1.03–2.29	
**Subject screened for PCa in the last 2 years**							
Any exposure	669	1.13	0.92–1.39		1.16	0.92–1.44	
Cumulative duration (years)				0.08			0.04
]0–15]	182	0.95	0.71–1.27		0.96	0.71–1.30	
]15–30]	269	1.19	0.91–1.55		1.18	0.89–1.56	
>30	218	1.23	0.93–1.62		1.33	0.99–1.78	
**5-year lag time**							
Any exposure	662	1.22	1.02–1.48		1.24	1.02–1.52	
Cumulative duration (years)				0.01			0.01
]0–15]	225	1.06	0.83–1.35		1.08	0.84–1.40	
]15–30]	308	1.32	1.05–1.66		1.31	1.03–1.66	
>30	129	1.34	0.99–1.82		1.42	1.04–1.96	
**Retired subjects**							
Any exposure	169	1.46	1.00–2.12		1.62	1.06–2.49	
Cumulative duration (years)				0.03			0.02
]0–15]	39	1.27	0.73–2.22		1.45	0.79–2.68	
]15–30]	66	1.40	0.88–2.25		1.51	0.89–2.58	
>30	64	1.72	1.05–2.82		1.96	1.12–3.43	
**Self-respondents**							
Any exposure	660	1.25	1.04–1.52		1.27	1.04–1.56	
Cumulative duration (years)				0.01			< 0.01
]0–15]	179	1.07	0.82–1.38		1.08	0.82–1.42	
]15–30]	270	1.35	1.06–1.72		1.33	1.03–1.72	
>30	211	1.32	1.02–1.71		1.39	1.06–1.81	

*OR, odds ratio; CI, confidence interval; PCa, prostate cancer*.

*^a^Adjusted for age, ancestry, first-degree family history of PCa, family income, education, marital status, body mass index, type 2 diabetes, depression treated with medication, alcohol consumption, smoking, physical activity at work, and frequency of fruit and vegetable intake*.

*^b^Polynomial logistic regression model. Reference category: subjects reporting no perceived work-related stress*.

Odds ratios were largely unchanged in analyses restricted to subjects with no missing data on workplace stress (data not shown), to subjects recently screened for PCa or to retired subjects (Tables 4 and 5). Results were unchanged when excluding proxy respondents, representing less than 4% of subjects, or subjects treated for depression. Applying a 5-year lag time did not affect the results. Age at first exposure, delay since last exposure (</≥5 years), ever experience of night work and occupational socioeconomic class did not modify the association between perceived workplace stress and PCa (data not shown). When the threshold to stratify analyses on age was moved from 65 to 60 years, the association with workplace stress duration was slightly more pronounced among younger subjects.

## Discussion

### Main Results

Perceived workplace stress duration was associated with higher risk of PCa (high- and low-grade cancers) among men less than 65 years of age, but not among older men. Occupations described as stressful were most often among white-collar workers. The timing of exposure to stress at work (age at exposure, time between exposure and index date) did not influence risk estimates. Adjustment for socio-demographic and lifestyle factors had a marginal impact on findings.

### Comparisons with the Literature

#### According to Cancer Type

A number of studies have reported an association between stressful life events and breast cancer, with the overall evidence judged as unconvincing (24, 25). Work-related stress was also mostly studied in relation to risk of breast cancer, with conflicting results (7–9, 12, 26–28). However, hormonal response to stress (29, 30), work-related stress determinants (31), and the relative physiological (29) and psychological (32) impact of stressors from private and occupational life may differ between men and women (26, 29, 30). These findings justify the conduct of sex-specific studies of stress and health.

To our knowledge, only two studies analyzed PCa risk in relation to stress at work, where no association was observed. One was a meta-analysis of 12 European cohort studies (12). While the individual studies benefited from a detailed job strain assessment, it was conducted at baseline only (among subjects aged 17–70 years); job strain was infrequent, experienced by 9% of the 865 incident PCa cases. The other one was the Montreal Multisite Case-Control Cancer Study, using the same method as the current one to evaluate lifetime workplace perceived stress. This study was conducted in the early 1980s and was based on a small sample (400 PCa cases) (13).

#### According to Study Design

Case–control studies are more prone to recall bias. However, among studies investigating the relation between stress at work and cancer (any type), a similar proportion of studies observing a positive association was observed among case–control (3 out of 5) (26, 33–36) and cohort (3 out of 6) studies (7–9, 12, 27, 28).

#### According to Age

In our study, the relation between workplace stress duration and PCa risk was more pronounced among subjects less than 65 years of age. Two studies have focused on breast or colon cancer cases younger than age 65 at diagnosis, including one observing a positive association (7, 35). The absence of association among older subjects in our study may result from a non-differential recall bias (susceptible to become more pronounced with aging). Conversely, it might reflect a cohort effect, whereas stressors would differ depending on the era they occurred.

#### According to Workplace Stress Assessment

In previous studies focusing on cancer, work-related stress was assessed at only one or two time point(s), which hampered the investigation of the role of timing and duration of exposure. In view of recent arguments in favor of a reverse stressor–strain relationship (37) or of a role of age as a moderator (21), exploring the timing of the relation between work-related stress and cancer appears particularly pertinent. Our assessment of stress had the advantage of capturing changes in work stress over the entire career: 45% of subjects reported stress/non-stress or non-stress/stress sequences. Only 38.4% of exposed subjects reported having been stressed at work during more than 80% of their career. This suggests that a single stress measurement at baseline will result in substantial exposure misclassification and highlights the importance of measuring stress at multiple time points throughout the occupational life.

Stress at work has been mostly assessed in the past using the high demand-low control model developed by Karasek (15). Although it has been shown to be efficient in demonstrating a relation between chronic stress and cardiovascular (1, 2) or mental illness (3), this model might not capture cancer determinants of work-related stress. Our measure of workplace stress did not make assumptions about the stressors that could have an impact on cancer development. We found a greater proportion of white-collar workers among subjects ever stressed by their work. Our findings are in line with those of another study suggesting a role of job authority in the increased risk of breast cancer observed among higher-status occupations (9).

An advantage of measuring the individual’s perception of chronic stress is that it reflects subject susceptibility to stress health effects, whatever the stressor. Indeed the physiologic response to environmental stress depends on the individual’s perception of stress (6, 16). Only five studies assessed perceived stress in relation to cancer: three were based on the Copenhagen City Heart Study (10, 37, 38), two focused on PCa (10, 11), and one on breast cancer (26). None of them observed a positive association.

### Strengths and Limitations

#### Selection Bias

Response rates could have affected results if socioeconomic characteristics associated with workplace stress influenced subjects’ participation. However, according to Canadian census tract data for 2006, the rates for recent immigration, unemployment, low educational level, and low household income were similar in living areas of participants and non-participants, both among cases and controls, indicating that selection bias is not of major concern in the study. Besides, a healthy worker effect is improbable since participants were not selected according to their employment status at index date.

#### Detection Bias

Misclassification of PCa status due to under-detection is *a priori* limited in the present study. Indeed, it was set in a population with free access to healthcare and which was relatively uniformly and regularly screened for PCa at the time of subjects’ ascertainment. However, subjects with a lower socioeconomic status tended to be less frequently screened. Since subjects having experienced stressful jobs were more likely to be white-collar workers, the positive association between workplace stress and PCa risk could be in part due to higher levels of screening behavior among these subjects. However, adjusting on socioeconomic factors or on screening frequency did not result in substantial changes. Furthermore, similar results were observed in the analysis restricted to subjects recently screened.

#### Recall Bias

Recall bias may be at play in a case–control study, with cases being more prone to report stress. In a previous study investigating perceptions about the causes of breast cancer, nearly half of the women with breast cancer attributed their condition to mental or emotional factors (especially stress), whereas less than 30% of control women reported these factors to be causal (38). Such widespread belief could set the grounds for over-reporting of stress exposure by cases. However, the present study presents a number of attributes that may mitigate recall bias. The study was set among men; whether they would tend to ascribe PCa to stress has not been documented. Moreover, the PROtEuS project, presented as a “Study of the Environmental Causes of Prostate Diseases,” did not focus on stress, which represented only one question in a 2-h in-person interview eliciting information on a very large number of lifestyle and occupational factors. As well, the numerous and detailed questions asked about each job may have helped the subject in remembering past work environments and their context, regardless of their present situation. Finally, since depression has been shown to be associated with work-related stress (3), we used the report of a diagnosis of depression as an indicator of workplace stress less likely to be affected by a recall bias. We observed similar positive associations between workplace stress and depression among cases (OR = 1.31, 95% CI 0.96–1.78) and controls (OR = 1.44, 95% CI 1.05–1.99), which does not support an over-reporting of workplace stress among cases.

#### Workplace Stress Assessment

Our assessment of stress was based on a crude line of questioning, aimed at identifying the perception of stress associated with each job. Neither the degree nor the reasons (stressors) of reported workplace stress were elicited in our study. Our assessment went beyond job strain and could reflect other factors, such as job insecurity, hazardous environments, etc. We could document that stress was most often encountered in white-collars jobs, yet expected to benefit from more job control. These results are consistent with those of a Canadian survey, where workers stressed mainly about work were well-educated and held white-collar jobs (14). Among Quebec workers, self-rated workplace stress was positively associated with high psychological demand, but negatively associated with low skill discretion (39). Besides, the efficiency of Karasek’s demand-control model in predicting job strain has been challenged by recent findings suggesting that job control can act as a stressor among individuals low in emotional stability (40).

While the validity of the information on self-reported stress could not be verified, the interview-based job histories have been shown to be valid (41). The questionnaire used to obtain detailed work histories has been used extensively, notably in studies of lung, colon, and several other cancer types (42–46).

The detailed occupational history, including the question on perceived stress, was collected only for jobs held for 2 years and more, to keep interviews within a reasonable timeframe. However, this had a minimal impact as jobs lasting less than 2 years represented <4% of the overall career coverage in this population. Information about work stress was available for a subset of 24 jobs held for less than 2 years, allowing for some sensitivity analyses. Only four (16%) of these short jobs were reported to be stressful, representing a lower proportion than observed among longer jobs (36%). Besides, we found similar results in analyses restricted to subjects who never held a job lasting less than 2 years.

While unemployment or non-working time in other circumstances (subject was retired, sick or unavailable, homemaker, student, prisoner, refugee, or volunteer) could have been sources of stress related to work, these were not considered in the analyses as information about stress was only elicited for paid jobs. Perceived stress in at least one job was not associated with time since retirement.

#### Other Sources of Stress and Coping

We did not collect other sources of stress that may contribute to general day-to-day stress. Yet work is reported to be the main source of stress of daily life in Canadians, particularly among men, far ahead of financial concerns, lack of time, family matters, personal, and other issues (14). We did not account for personality characteristics and social support that may influence the way subjects cope with perceived stress, buffering adverse health effects of chronic stress (47). In a case–control study, high-effort coping was associated with increased PCa risk (11). Although modifying the relation between coping and PCa risk, neither social support nor perceived stress alone was associated with PCa risk in this study (11). Coping strategies may also be related to health seeking behaviors (i.e., attendance of screening examinations) and then to cancer diagnosis (48). We can hypothesize that personal characteristics such as anxiety could influence both stress perception and screening behavior. This could explain the association between workplace stress duration and PCa risk observed among men younger than age 65 but not among older men for whom screening for PCa is more common. However, whatever the age group considered, screening in the last 2 years was not associated with workplace stress duration among controls (adjusted on age, data not shown). Besides, including PCa screening frequency in multivariate models did not change substantially the results.

#### Other Methodological Issues

Another substantial strength of this study was its ability to take into account the potential confounding or effect modification by socioeconomic lifestyle and medical factors, albeit none, other than age, had a sizeable impact on findings. In the Danish Nurse Cohort, an interaction was observed between working nightshifts and job control on the association with hormone-related cancer risk in women (28). We did not observe an effect modification by night work when examining workplace stress in relation to PCa risk in our study.

Finally, this study is the largest to date to investigate the role of work-related stress in PCa risk and to consider PCa aggressiveness.

### Possible Biological Pathways

Stress leads to the activation of the sympathetic nervous systems and the hypothalamic–pituitary–adrenal axis, inducing secretion of catecholamines and glucocorticoids (6). Persistent activation of these neuroendocrine pathways can downregulate various functions of the cellular immune response, by decreasing activities of mediators involved in immune surveillance of tumors or by promoting genomic instability (49, 50). As well, cortisol has been shown to stimulate the growth of androgen-independent PCa cells (51). Finally, the relation between chronic stress and testosterone levels observed in several studies (52, 53) suggests that stress-induced hormones could contribute to PCa development.

## Conclusion

Unlike ours, previous studies did not observe an association between workplace stress and PCa. The inability of previous investigations to evaluate the role of exposure duration and to capture changes in stress levels over time, which were documented in the current study, could explain the divergent findings. Another issue is statistical power, as previous studies have included much fewer stress-exposed cases than ours. Our results suggest that perceived work-related stress may play a role in PCa development when experienced over a prolonged period. Stress was more often reported in white-collar jobs, and appeared to be essentially related to PCa risk diagnosed at a younger age.

## Ethics Statement

This study was approved by the Ethics Committees of the following institutions: Institut national de la recherche scientifique, Centre de Recherche du Centre Hospitalier de l’Université de Montréal, Hôpital Maisonneuve-Rosemont, Hôpital Jean-Talon, Hôpital Fleury, and Hôpital Charles-LeMoyne. The study was carried out in accordance with their recommendations. All subjects gave written informed consent in accordance with the Declaration of Helsinki.

## Author Contributions

AB-L conducted the data analysis and prepared the manuscript, M-EP led the conception, design and, data acquisition of the PROtEuS study. AB-L, M-CR, and M-EP contributed to the interpretation of the data and critical revision of the manuscript. All authors read and approved the final version to be published.

## Conflict of Interest Statement

The authors declare that the research was conducted in the absence of any commercial or financial relationships that could be construed as a potential conflict of interest.

## Abbreviations

PCa, prostate cancer; OR, odds ratio; IARC, International Agency for Research on Cancer; PROtEuS, Prostate Cancer & Environment Study; CCDO, Canadian Classification and Dictionary of Occupations; PSA, prostatic specific antigen; DRE, digital rectal exam.
